# Mitochondrial-Linked *De Novo* Pyrimidine Biosynthesis Dictates Human T-Cell Proliferation but Not Expression of Effector Molecules

**DOI:** 10.3389/fimmu.2021.718863

**Published:** 2021-11-24

**Authors:** Marlies J. W. Peeters, Pia Aehnlich, Adriano Pizzella, Kasper Mølgaard, Tina Seremet, Özcan Met, Lene Juel Rasmussen, Per thor Straten, Claus Desler

**Affiliations:** ^1^ National Center for Cancer Immune Therapy, Department of Oncology, University Hospital Herlev, Copenhagen, Denmark; ^2^ Center for Healthy Aging, Department of Cellular and Molecular Medicine, University of Copenhagen, Copenhagen, Denmark; ^3^ Department of Immunology and Microbiology, Inflammation and Cancer Group, University of Copenhagen, Copenhagen, Denmark

**Keywords:** T-cell metabolism, T-cell activation, pyrimidine *de novo* synthesis, mitochondrial respiration and oxidative respiration, immunosenescence and exhaustion

## Abstract

T-cell activation upon antigen stimulation is essential for the continuation of the adaptive immune response. Impairment of mitochondrial oxidative phosphorylation is a well-known disruptor of T-cell activation. Dihydroorotate dehydrogenase (DHODH) is a component of the *de novo* synthesis of pyrimidines, the activity of which depends on functional oxidative phosphorylation. Under circumstances of an inhibited oxidative phosphorylation, DHODH becomes rate-limiting. Inhibition of DHODH is known to block clonal expansion and expression of effector molecules of activated T cells. However, this effect has been suggested to be caused by downstream impairment of oxidative phosphorylation rather than a lower rate of pyrimidine synthesis. In this study, we successfully inhibit the DHODH of T cells with no residual effect on oxidative phosphorylation and demonstrate a dose-dependent inhibition of proliferation of activated CD3^+^ T cells. This block is fully rescued when uridine is supplemented. Inhibition of DHODH does not alter expression of effector molecules but results in decreased intracellular levels of deoxypyrimidines without decreasing cell viability. Our results clearly demonstrate the DHODH and mitochondrial linked pyrimidine synthesis as an independent and important cytostatic regulator of activated T cells.

## Introduction

Following antigen-mediated activation of T cells, a metabolic switch occurs that increases glycolysis in order to support the ensuing clonal expansion with copious amounts of ATP and cellular building blocks (1–3). During this expansion, mitochondrial oxidative phosphorylation (OxPhos) is not replaced but rather supplemented by an increase in glycolysis (4, 5). Indeed, there is a MTORC1-mediated increase of mitochondrial mass and mitochondrial DNA levels within the first hours of activation (5, 6). This results in higher rates of OxPhos and overall bioenergetics of physiologically activated T cells (4). Functional OxPhos is important for T-cell responses following activation. An inhibition of the mitochondrial electron transport chain (ETC) responsible for OxPhos has been demonstrated to block proliferation and cytokine production of T cells (3, 5–9). Furthermore, decreased activity of OxPhos during the activation of T cells has been linked to the exhaustion phenotype observed in chronic viral infection (10, 11) and in the immunosuppressive microenvironment of a tumor (12, 13). Reduced OxPhos activity has also been linked to the inability of T cells of aged mice to respond to stimulation (14). Because of this, modulation of OxPhos has been suggested to improve T-cell activation in elderly and is investigated in combination with immunotherapies for the revitalization of exhausted T cells (12–14).

The requirement of a functional OxPhos for the activation of T cells is not fully understood, but it has been associated with production of ATP (3), production of mitochondrial reactive oxygen species (ROS) for intracellular signaling (9), change of lysosomal function (6), and production of substrates for one-carbon metabolism (14). Likely, also other processes are involved.

The enzyme dihydroorotate dehydrogenase (DHODH) links OxPhos to *de novo* pyrimidine synthesis (15). DHODH catalyzes the conversion of dihydroorotate to orotate in the fourth step of the *de novo* synthesis of pyrimidines. The enzyme is located in the inner membrane of the mitochondria (16) and its activity depends on a functional ETC, as dihydroorotate oxidation is linked to respiratory ubiquinone reduction (17). Impaired function of the ETC, due to hypoxia, inhibitors, or mutations of complex III and IV of the ETC, decreases activity of DHODH and inhibits thereby *de novo* synthesis of pyrimidines (18, 19).

In clinical trials, inhibitors of DHODH have been demonstrated to reduce severity of multiple sclerosis and rheumatoid arthritis, due to their immunosuppressive attributes (20, 21). Treatment of lymphocytes with the DHODH inhibitors leflunomide or the active metabolite of leflunomide, teriflunomide, has demonstrated cytostatic properties that can be reversed by the addition of the pyrimidines uridine and cytidine but not the addition of purine nucleosides (22–24). Inhibition of DHODH resulted in a decreased release of pro-inflammatory cytokines that could not be reversed by supplementation with uridine (24). Treating activated CD4 cells with leflunomide resulted in a decreased expansion of Th1 cells, while promoting Th2 cells both *in vitro* and *in vivo* (25). Recently, inhibition of the DHODH with teriflunomide was shown to result in an effective restriction of proliferation of murine CD4/CD8 T cells following antigen stimulation (20). This effect was co-occurring with a severe impairment of complex III of the electron transport chain, and the authors concluded that the effects of DHODH inhibition on T cells was due to an interference with OxPhos and aerobic glycolysis (20).

In this study, we show that an inhibition of DHODH impairs proliferation of activated human T cells. We establish that this effect is not due to interference with the activity of OxPhos and glycolysis, but rather due to reduced levels of pyrimidines. The cytostatic effect can be fully rescued upon exogenous uridine addition. This places mitochondrial-linked pyrimidine synthesis as an important cytostatic regulator of activated T cells.

## Materials and Methods

### PBMC Isolation and Stimulation

PBMCs from healthy donor buffy coats were isolated by gradient centrifugation and used immediately or cryopreserved for later use. PBMCs were cultured with αCD3/CD28-coated Dynabeads (Gibco, 1:1 bead-to-cell ratio) for 3 days in the presence or absence of 5 nM to 500 nM brequinar (Sigma-Aldrich) or 0.5 µM to 500 µM teriflunomide (Sigma-Aldrich) in X-VIVO 15 media (Lonza), supplemented with 5% heat-inactivated human AB serum (Sigma-Aldrich) and 50 U/ml hIL-2 (Proleukin). X-VIVO media was not further supplemented with nucleosides or nucleotides unless specifically mentioned. Stock brequinar was diluted in DMSO. In assay media, DMSO is diluted 10,000-fold or more. Addition of brequinar was demonstrated not to have an immediate effect on OxPhos (**Supplementary Figure 3G**). All cells were cultured in a humidified 37°C, 5% CO_2_ incubator. When mentioned, uridine was added in a concentration of 16 µM.

### Flow Cytometry

For proliferation assays, cells were labeled with proliferation dye CellTrace Violet (Invitrogen). For surface staining, single-cell suspensions were stained with the following fluorescently labeled antibodies: anti-CD3 (clone UCHT1), anti-CD8 (RPA-T8, both BD Biosciences), and anti-CD4 (SK3, SCBT). For intracellular staining, cells were incubated with Golgiplug (BD Biosciences) and anti-CD107a (clone H4A3, BD Biosciences) for 5 h prior to extracellular staining. After staining with the fluorescently labeled surface antibodies anti-CD3 (clone UCTH1, BD Biosciences) and Fixable Near IR Dead Cell Stain (ThermoFisher Scientific), cells were fixed and permeabilized using Intracellular Fixation and Permeabilization Buffer Set (eBioscience) and stained with the following fluorescently labeled antibodies: anti-tumor necrosis factor-alpha (TNFα, clone MAb11, BD Biosciences), granzyme B (GB-11, Thermo Fisher scientific), anti-interferon-gamma (IFNγ, 4S.B3), anti-perforin (B-D48), and anti-granulysin (DH2, all BioLegend). Sample acquisition was performed using the LSR II (BD Biosciences) or Novocyte Quanteon (ACEA Biosciences) and data were analyzed using FlowJo v10. Gating strategy is depicted in **Supplementary Figure 1A**. T-cell purity is depicted in **Supplementary Figure 1**.

### Cytokine Measurements

Levels of IFN-γ were measured in culture supernatants using enzyme-linked immunosorbent assays (ELISAs) according to the manufacturer’s instructions. Results were analyzed using Epoch plate reader (BioTek) and Gen5 Take3 software (v1.00.4, BioTek).

### Quantification of Intracellular dNTP Levels

Intracellular levels of dNTPs were measured in 3-day αCD3/CD28-stimulated T cells grown in the presence or absence of brequinar (50 or 100 nM). Samples were frozen in 60% methanol. Cellular dNTPs were extracted from 2 million PBMCs. Subsequently, steady-state levels of deoxyadenosine triphosphate (dATP), deoxycytidine triphosphate (dCTP), deoxyguanosine triphosphate (dGTP), and deoxythymidine triphosphate (dTTP) were determined using the DNA polymerase extension assay previously described (26).

### Measurements of Bioenergetics

The bioenergetics from human PBMCs were measured in the presence or absence of DHODH inhibition in real-time using an XF-96 Extracellular Flux Analyzer (Seahorse Bioscience, Agilent). αCD3/CD28-stimulated PBMCs were grown in the presence or absence of brequinar (50 or 100 nM) for 3 days prior to use. Equal cell amounts were then resuspended in Seahorse assay media (Seahorse Bioscience, Agilent), supplemented with 1 mM pyruvate, 2 mM glutamine, adjusted to pH 7.4, and subsequently seeded in a Seahorse 96-well plate using Cell-Tak adherent (Corning). Oxygen consumption rates (OCRs) and extracellular acidification rates (ECARs) were measured. Wells were consecutively treated with 1 µM oligomycin (Sigma), 0.3 µM carbonyl cyanide p-(trifluoromethoxy) phenylhydrazone (FCCP, Sigma), and 2 µM Antimycin A (Sigma).

### Statistical Analysis

**Figure 1:**
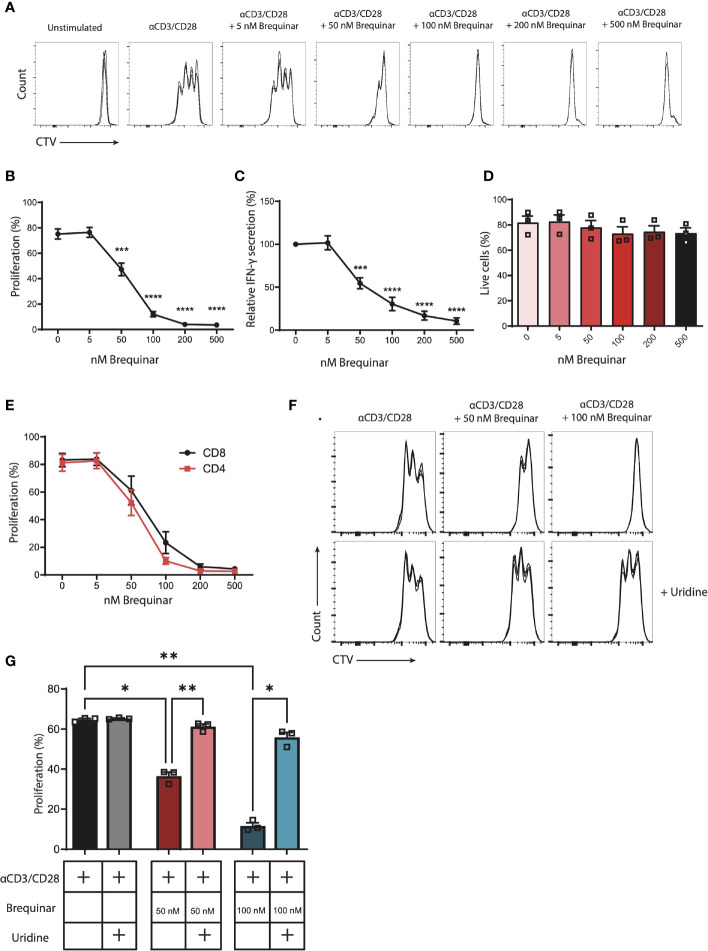
Proliferation of αCD3/CD28-stimulated CD3^+^ T cells is inhibited by brequinar treatment in a dose-dependent manner. Inhibition is reversed with uridine supplement. Human PBMCs were stained with a proliferation dye (CellTraceViolet, CTV) and activated for 3 days with αCD3/CD28 in the presence or absence of brequinar (5–500 nM). Proliferation and cell viability were measured by flow cytometry. **(A)** Representative histograms of technical triplicates of 1 donor. **(B)** Percentage of proliferating CD3+ T cells. **(C)** IFN-γ concentrations in culture supernatants. **(D)** Percentage of live cells. **(E)** Percentage proliferated CD4+ or CD8+ T cells. **(F)** Representative histograms of technical triplicates of one donor treated with brequinar and with or without 16 µM uridine. **(G)** Percentage proliferated CD3+ T cells with brequinar and with or without 16 µM uridine. Data are plotted as mean ± SEM (*n* = 3). **(D, G)** are dot bar graphs where each dot represents individual data points. *p < 0.05, **p < 0.01, ***p < 0.001, ****p < 0.0001.

**Figure 2:**
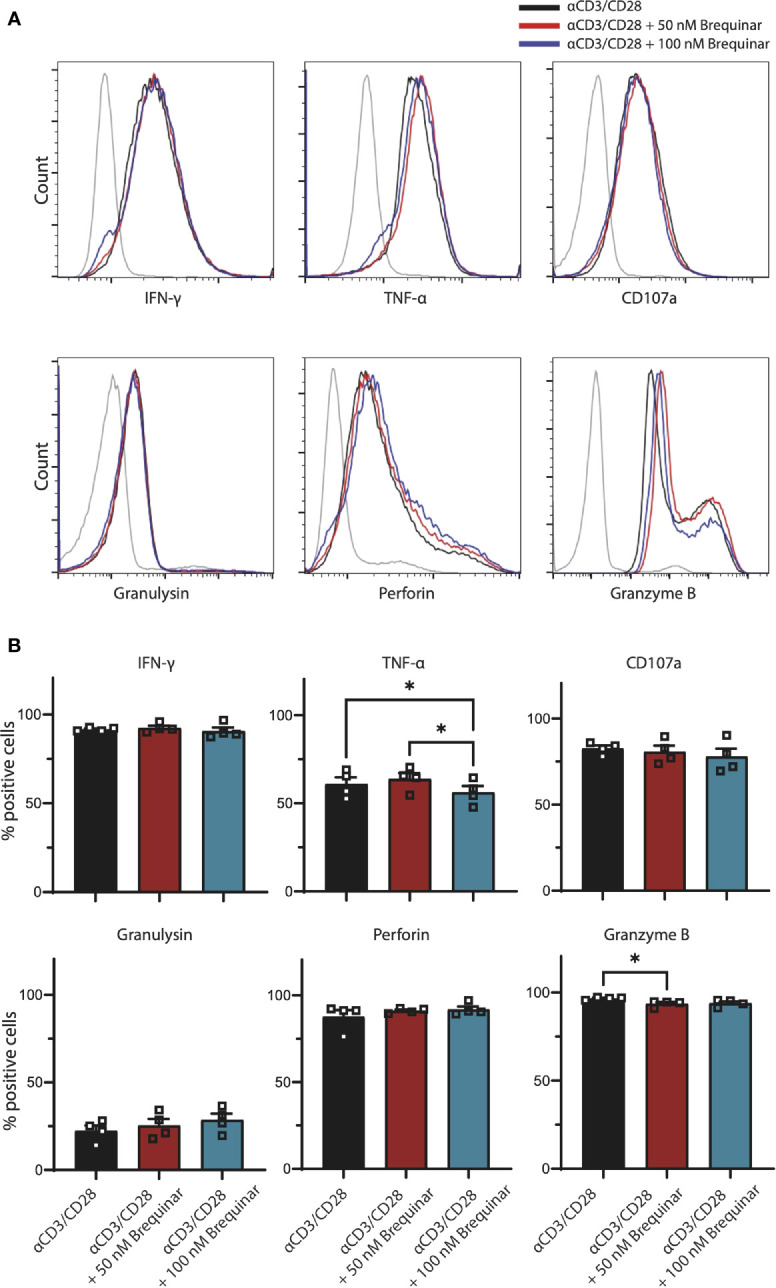
Expression of effector molecules by activated CD3+ T cells not affected by brequinar. Human PBMCs were activated for 3 days with anti-CD3/CD28 in the presence or absence of brequinar. **(A)** Representative histograms including unstimulated control (gray lines), activated T cells (black lines), and activated T cells with 50 nM (red lines) or 100 nM (blue lines) brequinar. **(B)** Percentage of CD3+ T cells expressing IFNγ, TNFα, CD107a, granulysin, perforin, and granzyme B, as measured by flow cytometry. Data are plotted as mean ± SEM (*n* = 4). **(B)** are dot bar graphs where each dot represents individual data points. *p < 0.05.

**Figure 3:**
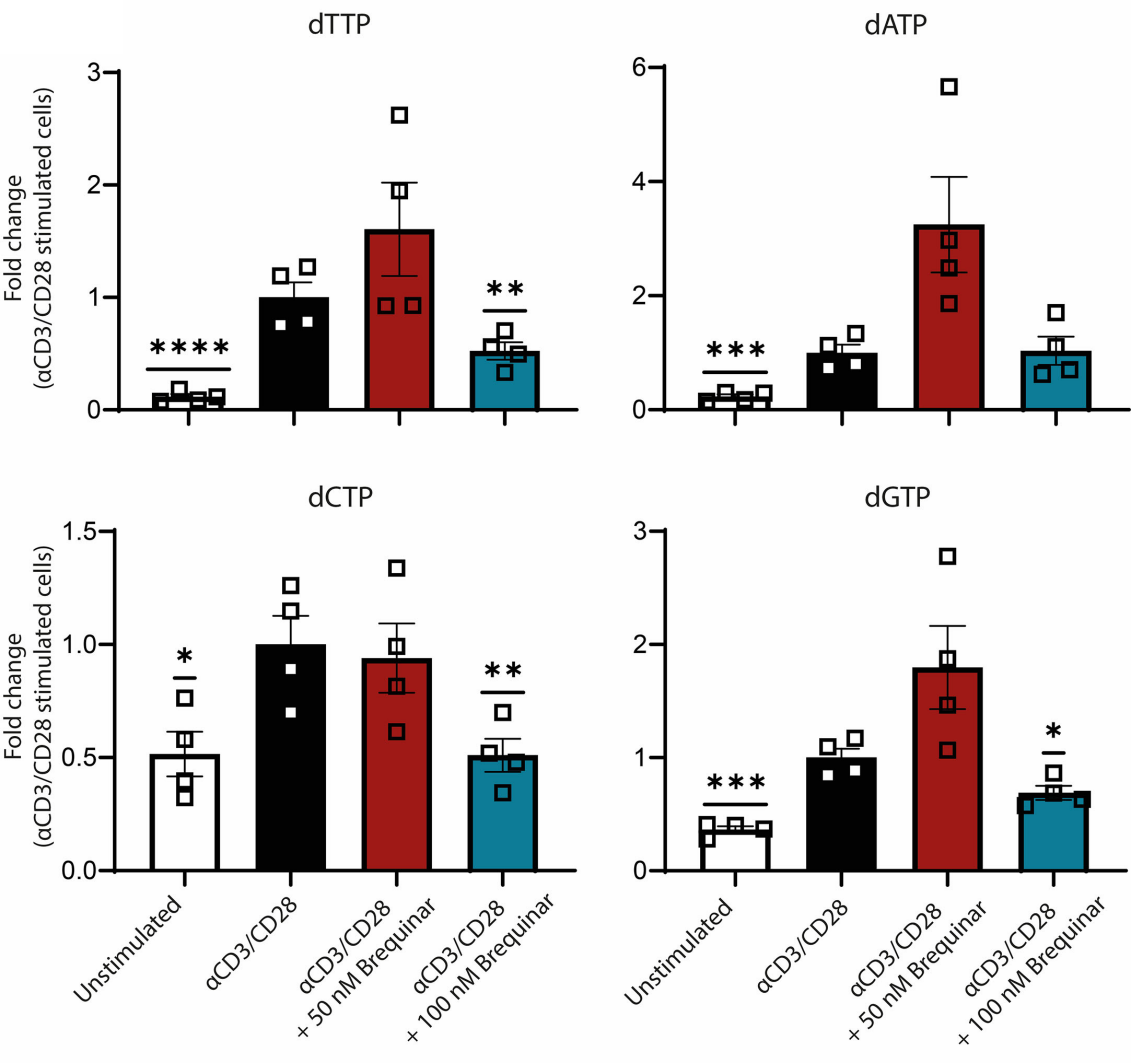
Nucleotide metabolism is inhibited by DHODH inhibition. Fold change of intracellular deoxyribonucleotide levels of αCD3/CD28-stimulated CD3^+^ T cells compared to unstimulated cells and stimulated cells treated with 50 and 100 nM brequinar. All levels are normalized to αCD3/CD28-stimulated CD3^+^ T cells. The levels of pyrimidines dTTP and dCTP and the purine dGTP is decreased at 100 nM of brequinar. Data are plotted as mean ± SEM (*n* = 4). Figure is a dot bar graph where each dot represents individual data points. *p < 0.05, **p < 0.01, ***p < 0.001, ****p < 0.0001.

**Figure 4:**
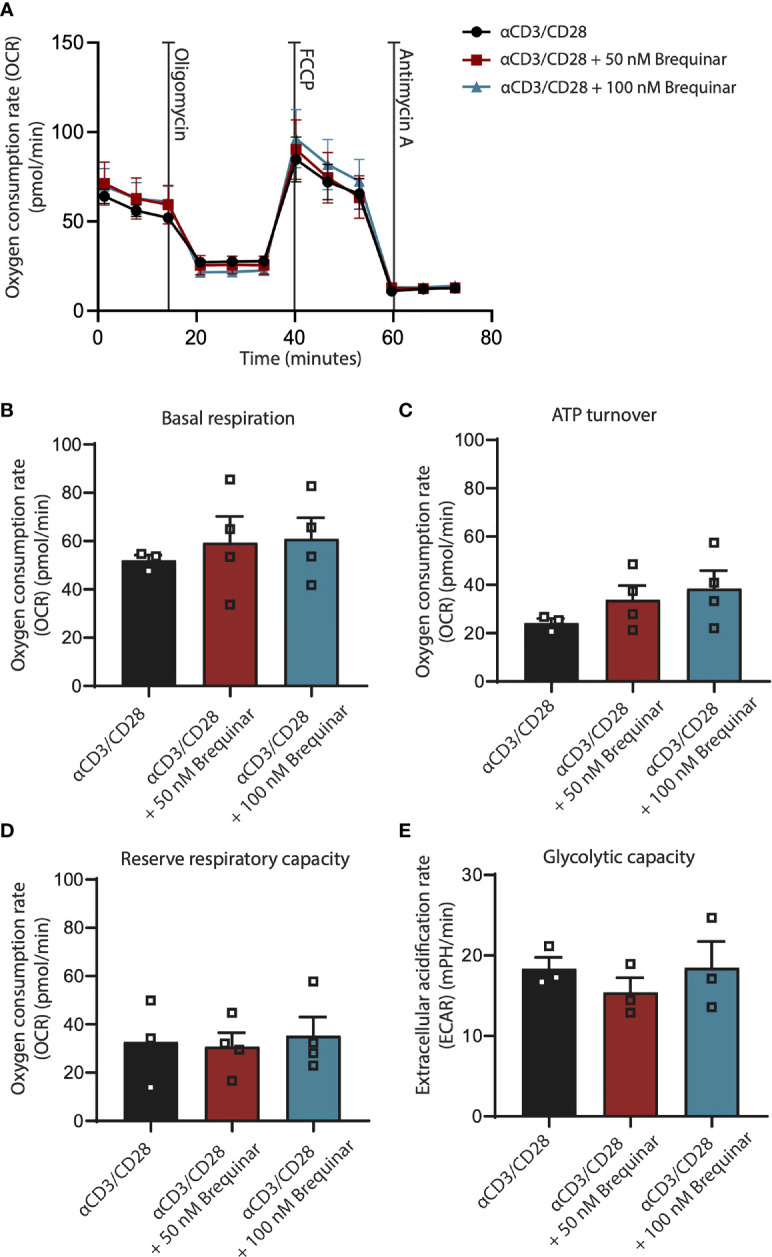
Respiration is not affected by inhibition of DHODH. Respiratory properties of αCD3/CD28-stimulated CD3^+^ T cells treated with 0, 50, and 100 nM brequinar. **(A)** Oxygen consumption rates of cells treated with oligomycin, FCCP, and antimycin A. **(B)** Basal respiration is determined as initial resting consumption of oxygen. **(C)** ATP turnover is measured as decrease of oxygen consumption after addition of oligomycin. **(D)** Reserve respiratory capacity is determined as the difference between basal respiration and respiration following FCCP addition. **(E)** Glycolytic capacity was measured after the addition of oligomycin. Data are plotted as mean ± SEM (*n* = 4). **(B, C)** are dot bar graphs where each dot represents individual data points.

Data are plotted as mean ± SEM. Comparisons between groups were analyzed with one-sample Student’s *t*-tests (**Figure 4**) and repeated-measures ANOVA with Tukey’s multiple comparisons tests (**Figures 1B**–**E**, **G**, **2B**, **3**) as appropriate. Data analysis was performed with GraphPad Prism (v9.00) software unless specified otherwise. Used statistical tests and number of biological replicates are indicated in the figure legends.

## Results

### Brequinar Inhibits Proliferation of Activated CD3^+^ T Cells in a Dose-Dependent Manner

Proliferation of CD3^+^ T cells was quantified following αCD3/CD28 stimulation. In the presence of 0–500 nM of the DHODH inhibitor brequinar, proliferation was inhibited in a significant and dose-dependent manner. At 50 nM of brequinar, proliferation was reduced to 63% of stimulated controls (*p* = 0.0002). At 100 nM of brequinar, proliferation was at 16% (*p* < 0.0001), while 200 and 500 nM of brequinar reduced proliferation to 5% (*p* < 0.0001) (**Figures 1A, B** and **Supplementary Figure 1**). In agreement, resulting media levels of secreted IFNγ also decreased in response to increased concentrations of brequinar (**Figure 1C**). When comparing the proliferation of CD4^+^ and CD8^+^ T cells, the proliferation of both subsets declined in a similar manner, demonstrating identical sensitivity towards brequinar for CD4^+^ and CD8^+^ T cells (**Figure 1E**). We were interested in determining whether the viability of the activated cells was affected by the brequinar treatment and found no significant decrease of viability when treating T cells with 5–500 nM brequinar (**Figure 1D**).

When brequinar treatment of T cells was supplemented with the pyrimidine nucleoside uridine, the cytostatic effect was completely reversed: No significant difference between the proliferation of uridine supplemented T cells treated with 50 nM or 100 nM brequinar and stimulated controls (*p* = 0.4672 and *p* = 0.2754, respectively) was observed (**Figures 1F, G**). Uridine did not increase proliferation in the cells not treated with brequinar (*p* = 0.9296) (**Figure 1G**). As a control, we treated cells with 0–500 µM teriflunomide (**Supplementary Figures 3A, E**). Teriflunomide also inhibited proliferation in a dose-dependent manner, where relative proliferation was significantly reduced to 81% at 10 µM of teriflunomide and 62% at 50 µM of teriflunomide. At higher concentrations, proliferation was almost completely stalled (**Supplementary Figure 3A**).

### Brequinar Treatment Does Not Impair Synthesis of CD3^+^ T-Cell Effector Molecules

To further evaluate the effect of brequinar treatment on general functionality of αCD3/CD28-stimulated CD3^+^ T cells, we quantified the levels of the pro-inflammatory cytokines IFNγ and TNFα, degranulation marker CD107a, as well as the cytotoxic molecules granulysin, perforin, and granzyme B in CD3^+^ T cells with intracellular staining (**Figure 2**). When comparing T cells treated with brequinar for 3 days with stimulated controls, we could only determine discrete changes in the expression of effector molecules. For T cells treated with 50 nM brequinar, only a 3% decrease of Granzyme B was significantly different from the profile of untreated cells (*p* = 0.0280). For T cells treated with 100 nM brequinar, only a 7.7% decrease of TNFα was significantly different (*p* = 0.0312). TNFα expression also differed between 50 and 100 nM brequinar treatment, the lower concentration showing a 13% increase in TNFα expression compared to the higher concentration (*p* = 0.0196) (**Figure 2**). All differences were determined on CD3^+^ T cells and by using unstimulated cells as a gating control. Even though statistically significant differences in effector molecule expression were observed, it should be noted that these changes are marginal and are highly unlikely to be of relevance in a biological setting. Comparison by MFI did not reveal any biologically relevant changes in cytokines or cytotoxic molecules either (**Supplementary Figure 2**). Thus, brequinar treatment did not seem to greatly impact the upregulation of effector molecules following TCR activation, which could mean that T cells retain their functional capacity.

### Brequinar Treatment Lowers Pyrimidine Deoxyribonucleotide Levels

We were interested in determining the inhibitory effect of brequinar on the DHODH and on the subsequent pyrimidine steady-state levels. Deoxyribonucleotides were measured as representative for the overall impact on nucleotide levels (15). As expected, we saw a 2- to 10-fold increase in deoxyribonucleotides following stimulation (**Figure 4A**) to 2.7, 1.1, 0.3, and 0.3 pmol/1 × 10^6^ cells for dTTP, dATP, dCTP, and dGTP, respectively. In stimulated T cells treated with 50 nM brequinar, we saw no difference in deoxyribonucleotide levels when compared to stimulated controls (**Figure 4A**). For T cells treated with 100 nM brequinar, the intracellular levels of the pyrimidines dTTP and dCTP were significantly decreased to 52% and 51% of stimulated controls, respectively (*p* = 0.0084 and *p* = 0.0068), while dGTP levels were also decreased, albeit to a smaller degree than the pyrimidines—69% of stimulated control (*p* = 0.0154) (**Figure 4A**). Deoxyribonucleotide levels are cell cycle-specific, and a cell cycle arrest will result in decreased levels of all deoxyribonucleotides. However, there was no significant difference between the dATP levels of T cells treated with 100 nM brequinar, when compared to stimulated controls (*p* = 0.8988). As the effect of brequinar treatment therefore predominantly affects the pyrimidines, we believe that the impact on nucleotide levels is directly caused by the DHODH inhibition rather than by cell cycle arrest.

### Inhibition of the DHODH by Brequinar Does Not Impair Mitochondrial Respiration

To answer whether inhibition of DHODH by brequinar would impair mitochondrial respiration, we measured the OCR and the ECAR of αCD3/CD28-stimulated CD3^+^ T cells before and after treatment with the oligomycin and FCCP (**Figure 4A**). This allowed us to determine the markers of respiration, basal respiratory rate, ATP turnover, reserve respiratory capacity, and glycolytic capacity. Basal respiratory rate is a baseline measure of the rate of oxygen consumed by the stimulated T cells before addition of mitochondrial inhibitors (**Figure 4B**). ATP turnover is measured as the decrease in OCR after inhibition of the ATP synthase (**Figure 4C**). This decrease is relative to ATP produced by oxidative phosphorylation. The reserve respiratory capacity is a measure of a theoretical extra capacity to produce ATP as a response to an increased energetic demand (**Figure 4D**). Glycolytic reserve is the difference in ECAR in the presence and absence of an ATP synthase inhibitor. The glycolytic reserve is, therefore, a measure of the glycolytic capability of a cell to respond to an inhibition of oxidative phosphorylation (**Figure 4E**). For stimulated T cells treated with 50 nM of brequinar and with 100 nM of brequinar, we saw no differences for these four markers of oxidative phosphorylation, which clearly demonstrate that the concentrations of brequinar used in this study did not affect any parameters of oxidative phosphorylation. For concentrations of 500 nM of brequinar, effects on T cell purity were observed (**Supplementary Figure 1B**). For 10 µM of brequinar, less than 20% of T cells were viable (**Supplementary Figure 3F**).

In contrast, when treating cells with teriflunomide, there is a significant impact on OxPhos capabilities similar to what has been reported by others (20) (**Supplementary Figure 3E**). The impact of teriflunomide became evident at 10 µM, when especially the reserve respiratory capacity is decreased (**Supplementary Figure 3E**). At 200 µM of teriflunomide, an almost complete inhibition of proliferation was associated with an almost complete loss of OxPhos capabilities.

## Discussion

Impairment of OxPhos is known to be detrimental for T-cell responses following activation. However, the mechanism responsible is not fully elucidated. In this study, we demonstrate that the proliferation but not functional capacity of αCD3/CD28-stimulated T cells is reduced following DHODH inhibition by brequinar. A similar cytostatic effect on activated T cells due to DHODH inhibition has been described (23, 24, 27, 28), but recently, these results were argued to be the result of a drug-induced modulation of OxPhos and aerobic glycolysis (20, 29, 30). Silencing of DHODH has been demonstrated to have little or no effect on OxPhos in several studied cell models [reviewed in (31)], and in breast cancer and melanoma cells, less than 5%–10% of ATP produced by OxPhos could be attributed to DHODH (32). We clearly show that this inhibition is unrelated to parameters of OxPhos. Our data thus demonstrate that an isolated inhibition of the OxPhos-linked pyrimidine synthesis has the ability to regulate proliferation of αCD3/CD28-stimulated T cells.

There is an intimate link between OxPhos and DHODH-mediated pyrimidine synthesis. It has been demonstrated that the activity of the DHODH is dependent on the activity of OxPhos (17). Therefore, inhibitory modulations of the OxPhos are reflected in the activity of the DHODH. Conversely, inhibition of the DHODH has been related to inhibition of the OxPhos in several studies (20, 29, 30). It should be noted that in these studies, leflunomide or teriflunomide, the activated metabolite of leflunomide, was used. In our setup, teriflunomide also had cytostatic effects and disrupted OxPhos capabilities of the T cells already at lower concentrations, where proliferation was only mildly affected, indicating a possible correlative relationship. However, leflunomide and teriflunomide are known to have pleiotropic effects, and in one instance, teriflunomide was found to directly target the ATP synthase of the ETC (29). Brequinar is a potent DHODH inhibitor brequinar (33) which display long-lasting retention in the mitochondria (34). When we used brequinar instead of teriflunomide, we demonstrated clear cytostatic effects of the drug, while confirming that none of the multiple measured parameters of OxPhos or glycolysis were affected. Furthermore, a supplementation with the nucleotide analog uridine completely restored the proliferation of activated T cells. This clearly demonstrates that an inhibition of OxPhos by teriflunomide is due to pleiotropic effects, rather than being a consequence of an inhibited DHODH.

It is well established that an inhibition of OxPhos negatively impacts proliferation and cytotoxicity of activated T cells (3, 5–9). By differentiating the effect of a DHODH inhibition from a general OxPhos inhibition, we can therefore conclude that OxPhos-mediated pyrimidine synthesis has a regulatory role in T-cell activation.

The general importance of nucleoside and nucleotide synthesis for activated T cells, as well as other cell types, has been known for a long time. Loss-of-function mutation of the gene encoding CTP synthase 1 has in humans been shown to lead to impaired capacity of T and B cells to proliferate following activation (35). Proliferation is restored by expression of the wild-type version of the enzyme and by supplementing with exogenous cytidine and CTP (35). Pyrimidines as well as purines are necessary for DNA and RNA synthesis, but they also give rise to other metabolic intermediates in the form of UDP-glucose, UDP-galactose, UDP-glucuronic acid, and UDP-N-acetylglucosamine (UDP-GlcNAc), which, in turn, play critical roles in membrane lipid biosynthesis, glycosylation events, and others (15, 36).

Inhibitors of the *de novo* nucleoside synthesis, including methotrexate and mycophenolic acid, have been used as immunosuppressants (37, 38). In activated T cells, inhibition of the *de novo* pyrimidine synthesis has been shown to abrogate progression from early to intermediate and late S phase, whereas inhibition of purine synthesis leads to a G1 arrest and blocks progression from early to intermediate S phase of already proliferating T cells (38). An inhibition of both pyrimidine and purine synthesis results in apoptosis of activated T cells (38). This apoptotic response suggests that the inhibition of nucleosides and nucleotides leads to a deficiency of building blocks for DNA and RNA synthesis (15). In agreement, the same drugs used as immunosuppressive agents are also utilized as chemotherapeutics (37).

In this study, however, we do not observe an impact on viability of the activated T cells following brequinar treatment. Furthermore, neither deoxypyrimidine nor deoxypurine triphosphate levels are fully depleted at the concentrations of brequinar used. We do therefore not believe that the cytostatic effect of DHODH inhibition is due to an acute shortage of deoxynucleotide triphosphates for DNA synthesis.

The observed cytostatic effect of brequinar is primarily caused by an inhibition of the pyrimidine levels as a uridine supplementation completely rescues the T cells. Nevertheless, we do not see any significant change of the deoxyribonucleotide levels when treated with 50 nM brequinar, even though we see a reduction of proliferation to 62% of the stimulated control. Furthermore, previous studies of non-T-cell models have identified the brequinar concentrations needed to induce growth arrest to be in the micromolar range rather than in the nanomolar range as described in this study (39, 40).

We therefore believe that in T cells, the cytostatic effect of brequinar is not due to restricting levels of deoxyribonucleotides, but rather due to other metabolic intermediates synthesized by DHODH. Future work will reveal the OxPhos-related pyrimidine component responsible for the cytostatic effect on activated T cells, and hopefully, this will allow us a better understanding of the factors that contribute to mitochondrial-linked exhaustion phenotype and senescence in T cells.

## Data Availability Statement

The original contributions presented in the study are included in the article/**Supplementary Material**. Further inquiries can be directed to the corresponding authors.

## Author Contributions

MP, PS, and CD contributed to conception and design of study. MP and CD created the figures and wrote the manuscript. MP, PA, AP, KM, and TS conducted the assays. Critical revision of the article was done by PA, ÖM, LR, and PtS. All authors contributed to manuscript revision, read, and approved the submitted version.

## Funding

This study was supported by the Danish Council for Independent Research (Grant No. DFF-1331-00095B); Danish Cancer Society (Grant No. R72-A4396-13-S2); Nordea-Fonden, Novo Nordisk Foundation Challenge Programme NNF17OC0027812; The Danielsen Foundation; Dagmar Marshalls Fond; Else og Mogens Wedell-Wedellsborg Fond; AP Møller Fonden; Den Bøhmske Fond; KV foundation; Familien Erichsens Mindefond; Axel Muusfeldts Fond; Kong Christian den Tiendes Fond; Sven Wewers fond; Fabrikant Einar Willumsens Mindelegat; Købmand Sven Hansen og Hustru Ina Hansens Fond; Børnecancerfonden and Tømmermester Jørgen Holm og Hustru Elisa f. Hansens Mindelegat. PA received partial Ph.D. stipends from the Clinical Academic Group in Translational Hematology, part of Greater Copenhagen Health Science Partners, as well as from the Department of Immunology and Microbiology, University of Copenhagen, the Training Network for the Immunotherapy of Cancer funded by the EU (IMMUTRAIN; H2020 grant no.641549.

## Conflict of Interest

The authors declare that the research was conducted in the absence of any commercial or financial relationships that could be construed as a potential conflict of interest.

## Publisher’s Note

All claims expressed in this article are solely those of the authors and do not necessarily represent those of their affiliated organizations, or those of the publisher, the editors and the reviewers. Any product that may be evaluated in this article, or claim that may be made by its manufacturer, is not guaranteed or endorsed by the publisher.
