# Supplementary material for: Mitochondrial-Linked De Novo Pyrimidine Biosynthesis Dictates Human T-Cell Proliferation but Not Expression of Effector Molecules
Source: Front Immunol. 2021 Nov 24;12:718863. doi: 10.3389/fimmu.2021.718863 (PMC8652221; doi:10.3389/fimmu.2021.718863)
Supplement: Supplementary file 1 [file DataSheet_1.docx]

Supplementary Material

| 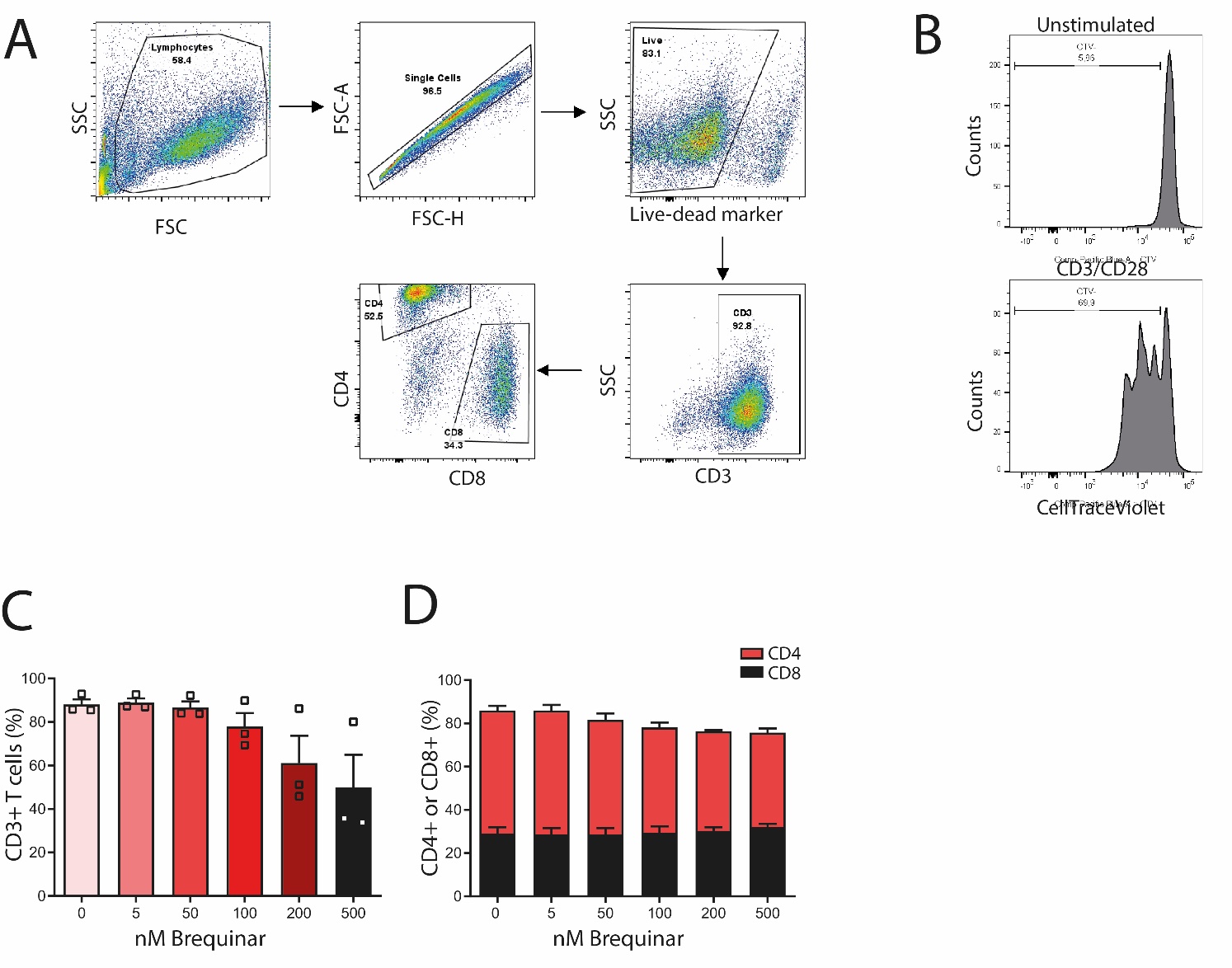 |
| --- |
| **Supplementary Fig 1** Human PBMCs were stained with a proliferation dye (CellTraceViolet, CTV) and activated for 3 days with anti-CD3/CD28 in the presence or absence of brequinar. Proliferation was measured by flow cytometry. (A) and (B) Gating strategy. (B) Percentage CD3+ T cells of live cells. (C) Percentage CD4+ or CD8+ T cells of CD3+ T cells. Data are plotted as mean ± SEM (n=3). |

| 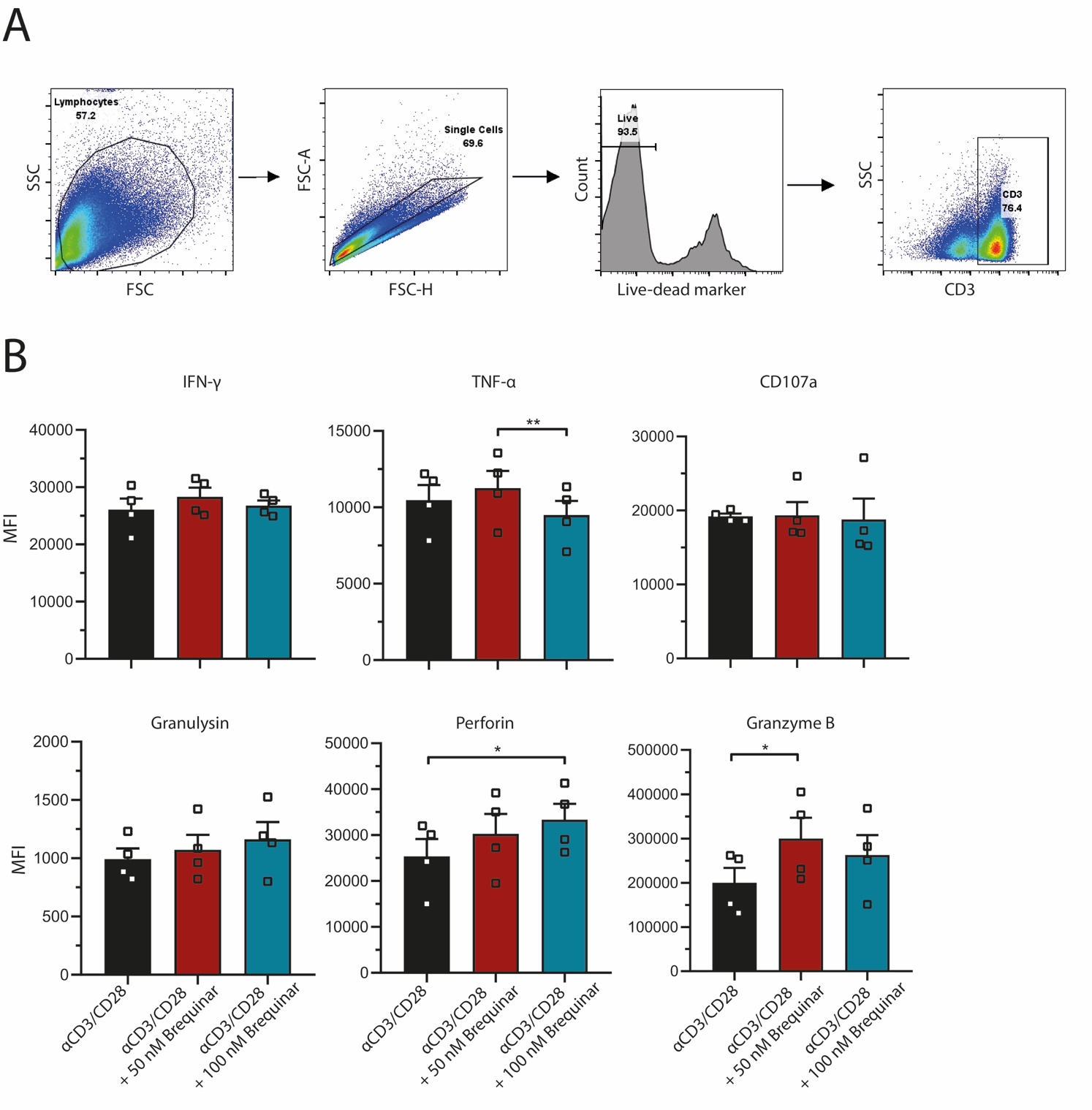 |
| --- |
| **Supplementary Fig 2** Human PBMCs were activated for 3 days with anti-CD3/CD28 in the presence or absence of brequinar, followed by intracellular staining measured by flow cytometry. (A) Gating strategy. (B) MFI of expression of IFN-γ, TNF-α, CD107a, granulysin, perforin, and granzyme B, in CD3+ T cells. Data are plotted as mean ± SEM (n=4). |
| 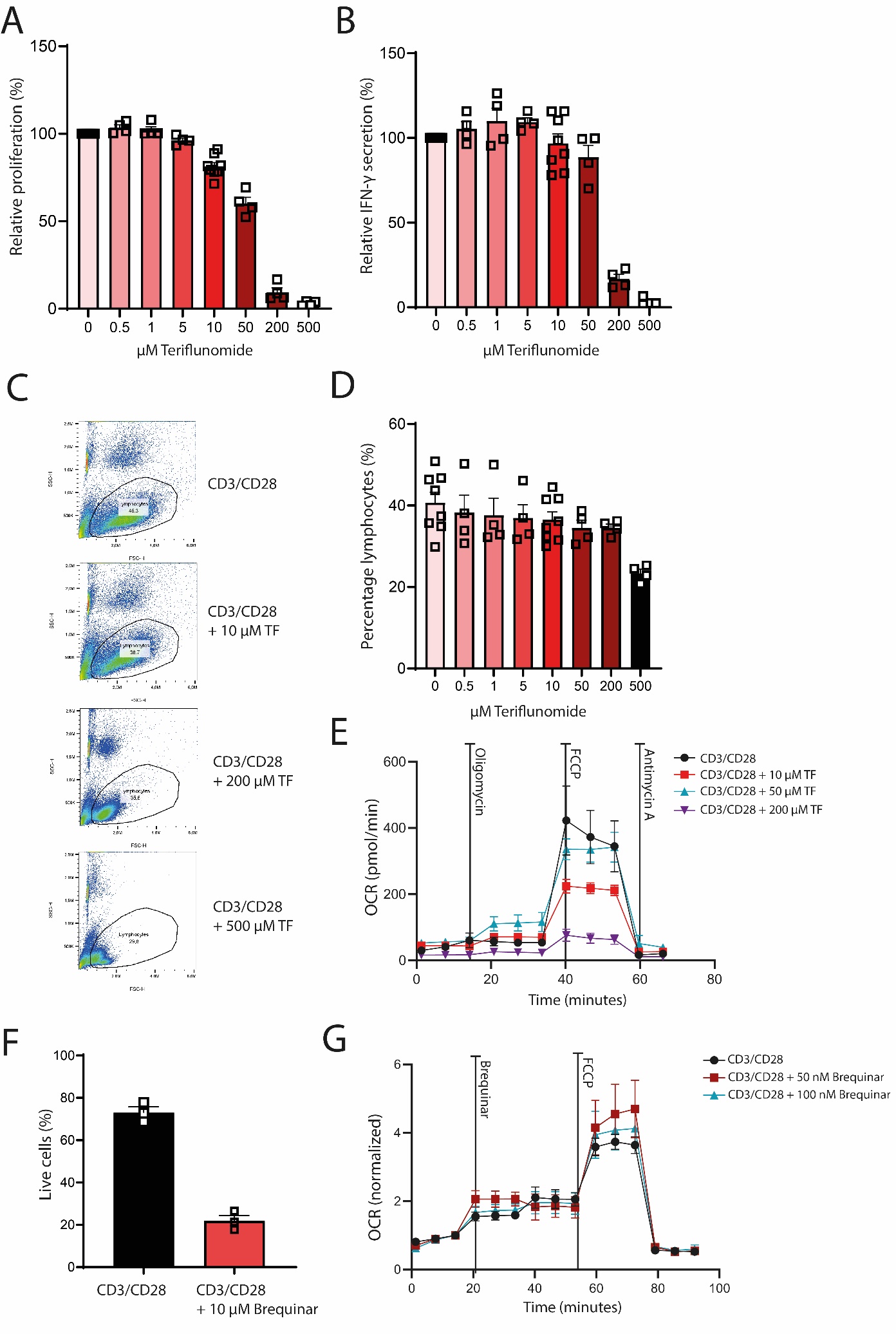 |
| **Supplementary Fig 3** Human PBMCs were activated for 3 days with anti-CD3/CD28 in the presence or absence of brequinar or teriflunomide. (A) proliferation was measured by flow cytometry using a proliferation dye (n=4 and n=8). (B) IFN-g concentrations in culture supernatants. (C) Representative dot plots of forward scatter and side scatter of cells cultured in the presence or absence of TF (teriflunomide) (n=4 and n=8). (D) Percentage lymphocyte sized T cells (n=4 and n=8). (E) Oxygen consumption rates of cells treated with oligomycin, FCCP and antimycin A (n=4). (F) Percentage of live cells when treated with 10 µM brequinar during three-day stimulation with CD3/CD28 (n=4). (F) Normalized oxygen consumption rate of three-day activated T cells treated with Brequinar and FCCP (n=4). Data are plotted as mean ± SEM. |
